# Development of a self-report measure to assess sleep satisfaction: Protocol for the Suffolk Sleep Index (SuSI)

**DOI:** 10.3389/fpsyg.2022.1016229

**Published:** 2022-10-26

**Authors:** Cleo Protogerou, Valerie Gladwell, Colin Martin

**Affiliations:** ^1^Institute of Health and Wellbeing, University of Suffolk, Ipswich, United Kingdom; ^2^Department of Psychology, University of Crete, Rethymno, Greece

**Keywords:** sleep satisfaction, sleep quality, development of self-report sleep satisfaction measure, study protocol, the Suffolk Sleep Index (SuSI), community-based participatory research

## Abstract

Good sleep is essential for health but there is no consensus on how to define and measure people’s understanding of good sleep. To date, people’s perceptions of a good night’s sleep have been, almost exclusively, conceptualized under the lens of sleep quality, which refers to *objective* characteristics of good sleep, such as such as ease and time needed to fall asleep, hours of sleep, and physical symptoms during sleep and upon awakening. A related, yet different construct, sleep satisfaction, refers to *perceived positive affect* about one’s sleep experience and has, to date, received little attention. This research focuses on sleep satisfaction, rather than sleep quality, and aims to develop a self-report measure to assess sleep satisfaction in an English adult population. As the measure will be developed in Suffolk, England, and its primary intended users are Suffolk community members, it is labelled the Suffolk Sleep Index (SuSI). The SuSI will draw from principles of community-based participatory research, following a seven-phase developmental process comprising literature review, interviews with Suffolk community members, synthesis of literature review and interview findings, pre-testing, pilot-testing, scale evaluation, and refinement. The present research will also investigate indices related to sleep satisfaction, including the community’s general health status, lifestyle factors and socio-economic status. The research will add to the limited, yet emerging body of research on perceived sleep satisfaction and its measurement. To our knowledge, a valid and reliable sleep satisfaction self-report measure has not been developed in the United Kingdom previously.

## Introduction

Sleep is essential for life, with good, healthy, sleep playing a critical role in maintaining optimal brain function, mood, and systemic physiology (Watson et al., 2015). However, disturbed sleep is a frequent complaint with more than half of adults in Western-based nations reporting short-term sleep disturbances, and between 15 and 20% of adults reporting long-term sleep disturbances (Ohayon, 2011; Manzar et al., 2018). Disturbed (or “troubled”) sleep is characterized by a broad range of physiologic, emotional, and behavioral symptoms, such as having difficulty falling asleep easily, having nightmares, waking up during the night and too early in the morning, not feeling refreshed upon awakening, and feeling sleepy and tired during the day (Cormier, 1990). In the short-term, disturbed sleep may lead to fatigue, low energy, physical pain, heightened response to stress, depression, anxiety, accidents, and deficits in cognition, memory and performance (Shelgikar and Chervin, 2013; Medic et al., 2017). In the longer term, disturbed sleep has been found to be implicated in weight gain, hypertension, dyslipidemia, metabolic syndrome, cardiovascular disease, type 2 diabetes, worsening of gastrointestinal disorders, increased risk of certain types of cancer, and all-cause mortality (Schernhammer et al., 2003; Shelgikar and Chervin, 2013; Sigurdardottir et al., 2013; Medic et al., 2017; Chattu et al., 2019).

The central role of sleep in overall health highlights the need to accurately conceptualize, define, and measure people’s understanding of a good and satisfying night’s sleep. To date, people’s perceptions of a good night’s sleep have been, almost exclusively, studied and measured under the lens of *sleep quality*, which refers to *objective* characteristics of good sleep, such as such as ease and time needed to fall asleep, hours of sleep, and physical symptoms during sleep and upon awakening (C. Kline, 2013). Sleep satisfaction is closely related, yet conceptually different to sleep quality and refers to perceived *positive affect* about one’s sleep experience (or how one *feels* about their sleep experience; Ohayon et al., 2018, 2019). The present research will focus on the understanding and measurement of perceived sleep satisfaction, rather than sleep quality, drawing from and extending a limited yet emerging body of research centering on sleep satisfaction. Given that research on sleep assessment to date has focused on sleep quality, we begin by providing background to the construct of sleep quality conceptualization and its measurement.

### Sleep assessment under the lens of *sleep quality*

While the term *sleep quality* has been extensively used in the published sleep literature as well as by health researchers, clinicians, and laypeople, sleep quality lacks a rigorous, agreed-upon, definition (C. Kline, 2013; Libman et al., 2016). Instead of being defined, sleep quality is being described by proxy quantifiable indices, especially the ease of sleep initiation, the ease and degree sleep maintenance, number of night-time awakenings, sleep quantity, and refreshment upon waking up (C. Kline, 2013). These indicators of sleep quality have been measured with objective instruments (e.g., physiological instruments like polysomnography and actigraphy; hardware devices like sleep apps), and with subjective instruments (e.g., questionnaires, sleep diaries). Both types of measures have advantages and disadvantages and may be seen a complementary (Ibáñez et al., 2018). Physiological measures, (i.e., polysomnography and actigraphy), are considered the most accurate sleep assessment measures, but they can be costly and—, require hardware, software, —and expertise to analyze and interpret (Toon et al., 2016). More recently, commercial devices (i.e., sleep *apps*) have been used by sleep researchers and laypeople for sleep assessment. Their public availability and low cost make these devices an attractive alternative to polysomnography and actigraphy, but most sleep apps do not grant access to the data collected or the scoring process, and furthermore, only a small number of sleep apps have been successfully validated against polysomnography and/or wrist actigraphy (Fino et al., 2020).

Given this complexity, sleep diaries and questionnaires are typically used as complementary to physiological measures, or as stand-alone measures. Despite their inherently subjective nature, the sensitivity of sleep diaries and questionnaires has been found to range between 73 and 97.7%, and the specificity between 50 and 96% (Ibáñez et al., 2018). Sleep questionnaires have been found to have good psychometric properties, with acceptable test–retest reliability, acceptable internal consistency, and acceptable convergent and discriminant validity with psychosocial and demographic variables (Fabbri et al., 2021). However, the questionnaires have been criticized in terms of their developmental method, scaling and scoring systems, and underlying factor structure (Yi et al., 2006; Raniti et al., 2018), thus undermining a key measurement cornerstone of validity. Lastly, extant subjective and objective sleep quality measures overwhelmingly focus on assessing the absence/presence of sleep *disturbances*, with an attempt to *quantify* rates of *disturbances*, suggesting that the measures may not assess sleep quality at all, but rather, the extent to which people experience sleep *problems* or *difficulty* (Libman et al., 2016; Ohayon et al., 2019).

### The present research: sleep assessment under the lens of *sleep satisfaction*

We echo emerging claims that sleep assessment methods have, essentially, focused on appraising sleep difficulty/disturbances, instead of sleep quality or satisfaction (Libman et al., 2016; Ohayon et al., 2019), and we align our thinking with Buysse’s (2014) call to approach good sleep in terms of *sleep satisfaction and sleep health,* instead of the absence of sleep difficulty and disturbance. We adopt Buysse’s (2014, p. 12) conceptualization of good sleep/sleep health as “…a multidimensional pattern of sleep-wakefulness, adapted to individual, social, and environmental demands, that promotes physical and mental well-being.” This *positive* approach to the experience of good sleep has several benefits. Firstly, it focuses on sleep patterns experienced by *all* people, regardless of whether they experience sleep disturbances. Second, it takes a *flexible* approach to people’s understanding of good sleep, acknowledging that it will vary as a function of time and context. Third, it suggests that good sleep is not discrete, or categorical, in nature (i.e., it cannot be neatly classed or measured in terms of *presence* versus *absence*), but is experienced on a *continuum* (e.g., good sleepers have a *graded* good sleep experience that may change across time).

We also note that the terms *sleep quality* and *sleep satisfaction* are used interchangeably in the literature (Buysse, 2014), and that research exploring similarities and differences between the two constructs in terms of their conceptualization and measurement is very limited (Ohayon et al., 2018). Still, we argue that while closely related, the sleep satisfaction and quality may have different meanings, with implications for assessment. To begin with, the terms satisfaction and quality are different, with *satisfaction—*denoting the degree to which one perceives one’s wants are being met, and *quality* referring to essential or distinctive characteristics, as well as to attributes of high grade, superiority, and excellence (Veenhoven, 2014). The present research focuses on satisfaction, as the degree to which one perceives one’s wants are being met, and is in line with Ohayon et al.’s (2018, 2019) pioneering work that has suggested disentangling sleep quality (and its measurement) from sleep satisfaction (and its measurement). For example, Ohayon et al. argued that, to date, sleep quality assessment has attempted to quantify a small number of *objective* sleep indicators, such as ease and time needed to fall asleep and maintain uninterrupted sleep, hours of sleep, and physical symptoms while being awake. This notwithstanding, sleep can be experienced as satisfying even in the absence of objective quality components; one may *feel* satisfied with only a few hours of sleep, while someone else may feel restless and anxious throughout long hours of sleep and thus unsatisfied. Under this approach, sleep satisfaction is conceptualized as perceived *positive affect* about one’s sleep experience. Critical components of sleep satisfaction include *how one feels* while they sleep, when they wake up in the morning, and during the next day; *how one feels* about the time and ease it takes them to sleep and resume sleep after being awakened during the night, the amount of sleep on weekdays and weekends; *how one feels* about their bedroom environment (e.g., noise, light, movement by others, temperature, bedding comfort); and one’s interest or motivation to experience satisfying sleep (Ohayon et al., 2018).

Positive perceptions (or positive appraisals) of one’s life experiences are strongly linked to better health outcomes, higher quality of life and sense wellbeing, and may even override the impact of objective biomarkers on one’s health and wellbeing (Conner et al., 2019). Although the intersection between sleep perceptions and subsequent health-related outcomes has received limited attention, there is evidence to suggest that subjective perceptions/appraisals of one’s sleep experience can impact health outcomes (Zavecz et al., 2020). For example, in an experimental study, Draganich and Erdal (2014) constructed positive or negative sleep perceptions by telling ‘normal’ sleepers that their sleep experience is below or above ‘average’, based on objective physiological measures. These constructed perceptions affected normal sleepers’ subsequent performance on memory and attention tests: those who were told they had experienced below average sleep quality the night before performed worse on cognitive tasks, and those who were told they had experienced above average sleep quality the night before performed better. Therefore, it can be argued that the ways one appraises their sleep experience can influence cognitive states in both positive and negative directions, suggesting a means of exerting control over—one’s health and cognition.

Ohayon et al. (2019) published the Sleep Satisfaction Tool (SST), a questionnaire that aims to measure sleep satisfaction and is targeted to the United States general population. Ohayon et al.’s work presents the first attempt to develop a sleep satisfaction (instead of quality) measure. We will build on this extremely limited, yet emerging, research to develop the first sleep satisfaction self-report instrument, the Suffolk Sleep Index (SuSI), targeted to a United Kingdom population. We opted for developing a new instrument, instead of adapting or validating the SST in our target population, for the following reasons. First, and in line with the principles of community-based participatory research that we employ, we will develop the SuSI with our community partners, targeting the needs and characteristics of our community members (we expand on our research approach below). Second, the SST does not include ambiance indicators of sleep satisfaction, but we argue that one’s sleep environment (e.g., characteristics of bedroom, bed and bedding; sleeping in urban or rural areas) can be instrumental in sleep satisfaction assessment. Third, we wish to conduct work towards developing an optimal and consistent scoring/scaling system. Fourth, we note that measures are incomplete without a user’s manual (Protogerou and Hagger, 2020) and wish to develop and test a user’s manual to accompany our instrument.

While we will be developing the SuSI, we will also be obtaining factors related to sleep satisfaction, including our population’s general health status, lifestyle—and socio-economic status.

## Materials and methods

### Approach to research

The SuSI will use community-based participatory research (CBPR), a collaborative approach to research that involves researchers, community members, and organizational representatives in varying degrees of partnership, with all partners contributing to knowledge, expertise, and decision-making (Israel et al., 1998; Wallerstein and Duran, 2010).^1^ CBPR has been used across health domains, including the development of behavioral health measures that aim to be contextually relevant (Garcia et al., 2008; Nielsen et al., 2014). These types of measures are also referred to as *targeted* because they are sensitive to the needs and characteristics of the population of interest, the local context, and the intended use of the measure scores (Kreuter and Skinner, 2000; Artino Jr et al., 2014). Furthermore, CBPR has been found to facilitate the implementation of health behavior interventions and policies, as it can bridge the gap between *what is known* from research and *the care that is delivered* by health systems (the so-called *know-do gap*) (Jull et al., 2017). We envision rapid implementation of the SuSI in the local community. Serving the local community and engaging with the local community in research is firmly embedded in our university’s vision and mission statement, as well as in our Equality, Diversity and Inclusion policy. Therefore, we have established a partnership to conduct CBPR with Suffolk Mind, a charity based in Felixstowe, Suffolk. Suffolk Mind offers a variety of mental health services to the wider Suffolk community with a focus on sleep health, and, historically, has been involved in community-based research. In partnership with Suffolk Mind, we have established a need for developing a novel self-report measure to assess perceived sleep satisfaction, targeted to the characteristics of the wider Suffolk community (a county located in the East of England, UK), which is our primary population of interest, but we will look to extend this beyond Suffolk after initial development of the measure.

### Design

The SuSI will be based on established best practices for developing and validating scales for behavioral health research (i.e., Beatty and Willis, 2007; Rattray and Jones, 2007; Gehlbach and Brinkworth, 2011; Artino Jr et al., 2014; Boateng et al., 2018). Specifically, the SuSI will be the outcome of seven developmental phases, explicated below and outlined in Table 1.

**Table 1:** Overview of seven-phase SuSI development process.

Phase	Purpose
1. Systematic literature Review.	To conceptualize sleep satisfaction and identify extant sleep satisfaction scales, items, response and scoring systems that may be used or adapted. To provide similarities and differences between sleep satisfaction and sleep quality.
2. Interviews with SuSI target population.	To establish how target population conceptualizes sleep satisfaction and whether this conceptualization matches the literature’s; (b) to provide evidence for the SuSI’s content and face validity.
3. Synthesis of literature review and interview findings.	To (a) ensure that SuSI’s sleep satisfaction definition/ conceptualization is both in line with prior evidence and understood by target population; (b) generate SuSI candidate sleep satisfaction domains and items; and (c) develop a user’s guide.
4. Pre-testing (cognitive interviewing and expert validation).	To ensure that (a) items are clear and relevant to sleep satisfaction/sleep satisfaction domains; (b) items are understood in the way they were intended to; and (c) user guide is understood and useful.
5. Pilot-testing (SuSI field administration).	To collect data to determine the SuSI’s dimensionality, factor/item structure, reliability and validity.
6. Scale evaluation.	To analyze pilot-testing data to determine SuSI, dimensionality, factor/item structure, reliability and validity
7. Refinement.	To finalize the SuSI and its user guide based on data collected from previous phases.

#### Phase 1. Literature review

The SuSI’s development will begin with a systematic literature review of published, peer-reviewed studies relating to sleep quality and satisfaction—to: (a) define/conceptualize sleep satisfaction and its domains, as well as factors facilitating/impeding sleep satisfaction; (b) identify conceptual and measurement similarities and differences between sleep satisfaction and sleep quality; (c) identify sleep satisfaction scales, items, response and scoring systems that may be of use; and (d) provide content and guidance for phase 2 of SuSI development.

#### Phase 2. Interviews

A series of one-on-one semi-structured interviews will be conducted with the SuSI’s primary intended users, i.e., Suffolk community members. The purpose of the interviews is to work towards establishing content and face validity of the SuSI sleep satisfaction domains and items. The interviews will provide information on (a) how the target population conceptualizes and understands sleep quality; (b) the extent to which the population’s conceptualization of sleep satisfaction matches the literature’s; and (c) sleep satisfaction components that are considered as crucially important by target population. These interviews will be based on the s*peak freely* procedure (Harvey et al., 2008). Specifically, participants will be first asked to talk freely for a few minutes about the characteristics of a night when they were satisfied with their sleep. Then, participants will be given a list of literature-derived sleep satisfaction components, as obtained in phase 1, and asked to indicate agreement/disagreement about the importance of each component, and to add components they consider important but not on the list. Interviewees will also be asked to complete a questionnaire comprising socio-demographic and health/lifestyle items, meant to describe participants and capture factors that have been found to be associated (in prior research) with sleep quality and satisfaction.

#### Phase 3. Synthesis of literature review and interview findings

The purpose of this phase is to (a) reconcile and consolidate the definition/conceptualization of sleep satisfaction and its domains by prior literature and target population; (b) generate SuSI candidate items that adequately describe sleep satisfaction using language that the target population can easily understand; and (c) develop an initial version of the SuSI user’s guide. The provision of a user guide with explanations of terms used and scoring strategies has been put forth as a necessary companion to self-report measures (Crowe and Sheppard, 2011; Protogerou and Hagger, 2020) but survey designers often omit it.

#### Phase 4. Pre-testing

The purpose of this phase is to test SuSI items with members of the target population and expert judges to further establish SuSI reliability and validity. Pre-testing will be accomplished by cognitive interviewing, followed by expert validation. *Cognitive interviewing* involves the administration of a draft of survey items to the target population and asking them to verbalize the cognitive processes involved in answering the items (Beatty and Willis, 2007). Cognitive interviews will employ a *concurrent verbal probing* procedure (Willis and Artino Jr, 2013), whereby members of the target population are asked a series of probe questions about their thought processes as they answer each question. Examples of probe questions include “what does the term X mean to you”? “Can you rephrase this question in your own words?” Cognitive interview data will be analyzed by so-called *predetermined coding* (Nápoles-Springer et al., 2006). Predetermined coding involves recording and tabulating common respondent “errors” (e.g., respondent does not know how to answer question; respondent requires clarification; asks to repeat question; respondent perceives item as redundant; respondent alters the meaning of item; respondent cannot recall information needed to answer question), and the frequency of errors. These error codes are *pre*determined because they reflect *typical* respondent errors are expected to be captured by the probe questions. Additional error codes may be generated during the interviews based on participants’ commentary and feedback. The information gathered from cognitive interviews will be used to modify/ improve candidate SuSI items, and to develop the shortlist of candidate items to be used in the next step of this phase (i.e., expert validation). In the context of self-report measure development, *expert validation* involves collecting data from expert judges to ensure that self-report items represent the construct being measured and that key items and/or construct domains have not been omitted (Polit and Beck, 2006). Specifically, we will use an *online expert consensus survey* to ascertain expert judges’ agreement or disagreement on the (a) *clarity, relevance/ representativeness* of shortlisted candidate items and *comprehensiveness* of whole scale; (b) the *appropriateness* of the scaling and scoring system; and (c) the *usefulness* of the user’s guide. We will follow Waggoner et al.’s (2016) best practice guidelines for (online) consensus research, that is, defining experts and consensus *a priori*, recruiting at least 11 experts, and conducting a comprehensive analysis of consensus data. Following extant guidelines on who may be considered an expert in a consensus panel (Yap et al., 2014; Jorm, 2015), we will recruit experts based on their involvement in relevant research and authorship of relevant publications, and aim for expert ‘diversity’ (e.g., from diverse geographical locations, professional ranks, genders, and age groups). We will adopt a conservative 80% criterion for acceptable agreement, in line with prior consensus research (Hasson et al., 2000; Keeney et al., 2006). Experts will be sent a content validation form on which they will rate the clarity, relevance, and representativeness of shortlisted candidate items, and the appropriateness of the scaling and scoring system. We will adapt and use Rubio et al.’s (2003) and Gehlbach and Brinkworth’s (2011) expert content validation templates, including closed- and open-ended questions. Expert consensus data will be used to further refine the SuSI prior to its distribution.

#### Phase 5. Pilot-testing

During pilot testing, the SuSI will be distributed to members of the target population with the purpose to collect data to further establish its factor and item structure, reliability, and validity. Pilot-testing is aptly referred to as the *dress-rehearsal* of survey administration, implemented to determine whether problems exist that need to be addressed prior to putting the survey in the field (Presser et al., 2004). The survey will be developed and administered online with QuestionPro software.^2^ As trustworthy test–retest reliability indexes should be, ideally, separated by at least a three-month gap (P. Kline, 2013), we intend to implement a prospective design with two survey administration points after the first one (about 30 and 90 days after first administration). To ensure participant anonymity and confidentiality, we will follow the Anonymous Repeated Measurements *via* Email (ARME) procedure developed by Carli et al. (2012). The ARME is a 7-step procedure that may be used in online prospective survey designs, outlined with a hypothetical participant below:

1. Participant 1 is asked whether they are interested in participating in the SuSI online survey. Upon confirmation, Participant 1 receives the random participant ID ****.
2. The *ad hoc* email address ****@mail.com is created by the researcher.
3. Participant 1 accesses the *ad hoc* email, changes the password and sets the account to forward all emails their regular email address.
4. Any information stating that Participant 1 received participant ID **** is destroyed.
5. Data collection: The researcher sends an email to ****@mail.com, in which they instruct Participant 1 to access and complete the online SuSI questionnaire.
6. Participant 1 receives this email at their regular email address after it has been forwarded from ****@mail.com. Participant 1 accesses and completes the questionnaire at both time points, identifying themselves only through their participant ID, ****.
7. Participant responses are safely stored in the study server and can be matched between the two time points.

During pre-testing, survey respondents will also be asked to complete the socio-demographic and health/lifestyle questionnaire (the one that is to be used in phase 2).

#### Phase 6. Scale evaluation

The purpose of this phase is to analyze data obtained from pilot-testing to further determine SuSI reliability, validity and factor/item structure. Specifically, we intend to ascertain the SuSI’s internal factor structure/dimensionality; internal consistency of item scores; test–retest reliability; convergent validity; discriminant validity; predictive/criterion validity; and known-groups validity. This phase will also entail a separate item reduction analysis, to ensure that only the most parsimonious, functional, and internally consistent items are ultimately included in the final version of the SuSI.

#### Phase 7. Refinement

This phase will produce the final layout of the SuSI drawing from findings obtained in phase 6. The SuSI user guide will also be finalized, considering findings and “lessons learned” across all developmental phases.

### Sampling and recruitment

The SuSI’s target population are adult Suffolk community members. In addition, international sleep researchers will be participants on the study’s expert validation/ consensus element. To elaborate:

All residents of the Suffolk region who are adults (≥ 18 years old) will be eligible to participate. The study will be advertised through our partner’s (Suffolk Mind) email account, social media platforms, and public board announcements, and the Suffolk University’s website. Experts in the field of sleep (satisfaction) research will be identified through their relevant research and publications. They will be invited *via* email to our online consensus survey, to judge the clarity, relevance, and representativeness of shortlisted candidate SuSI items, the appropriateness of the scaling and scoring system, and the usefulness of the user’s guide.

### Sample size calculation

Qualitative interview sample size is typically determined *via* saturation, that is the point where no or very few new insights emerge (Bowen, 2008). Saturation may be reached with a small sample, e.g., just six participants (Morse, 2000), or with larger samples, ranging between 20 and 30 participants (Creswell, 2002). For expert consensus studies, a recommendation of a minimum of 10 judges has been suggested (Waggoner et al., 2016). Completion rates on online consensus surveys tend to be very high – nearing 100% (e.g., Protogerou and Hagger, 2020), but response rates are approximately 33% (Nulty, 2008); we therefore intend to invite 40 experts, approximately, to reach the minimum recommendation of 11 experts. Survey sample sizes will vary, depending on the survey’s purpose and statistical analyses to be conducted. Survey pilot-testing typically requires about 300 respondents (MacCallum et al., 1999). Reliability testing can be accomplished with smaller sample, e.g., 100 respondents approximately or ≤ 100 for test–retest reliability estimation (Boateng et al., 2018).

### Data analyses

Prior evidence and interview data will be synthesized *via* inductive and deductive content analyses following processes described by Elo and Kyngäs (2008). Content analyses are systematic ways of analyzing data aimed at producing a “…condensed and broad description of the phenomenon, and the outcome of the analysis is concepts or categories describing the phenomenon” (Elo and Kyngäs, 2008, p. 108). *Deductive* content analyses are “top-down” or “classification from above” approaches to data, whereby the focus is on summations or summaries of data, and the identification of the most ‘popular’, ‘prominent’, and ‘frequent’ patterns of data (Elo and Kyngäs, 2008). Deductive content analyses are typically conducted when prior evidence exists. Prior literature will be analyzed deductively to generate an evidence-based definition/conceptualization of sleep satisfaction and comprehensive list of SuSI candidate sleep satisfaction domains and items. *Inductive* content analyses are “bottom-up” or “classification from below” approaches to data, whereby the focus is on going beyond summarizing data and identifying prominent data patterns to *interpreting* data patterns (Elo and Kyngäs, 2008). Participant-generated sleep satisfaction definitions, conceptualizations and candidate sleep satisfaction domains and items will be derived *via* inductive content analysis. Combining deductive and inductive data analysis approaches to generate new survey items is considered as “best practice” (Boateng et al., 2018).

Expert consensus data will be analyzed through quantitative and qualitative approaches. Data from closed-ended questions will be used to calculate inter-rater agreement coefficients with respect to the clarity, relevance, and representativeness of shortlisted candidate items, the appropriateness of the scaling and scoring system. Open-ended questions will be content-analyzed, following Elo and Kyngäs (2008). Pilot-testing data will be analysed with exploratory factor analyses, correlation and regression tests, and differences between groups tests.

### Ethics statement

The present study has been approved by the University of Suffolk Research Ethics Committee (approval ID # RETH(S)21/051). In summary, ethics procedures will include the provision of detailed information, informed consent, and debriefing sheets at all research phases.

## Discussion

Good, satisfying sleep is a prerequisite for health and wellbeing but, currently, there is no consensus on how to conceptualize, define, and measure perceived sleep satisfaction. People’s perceptions of good sleep have been, almost exclusively, assessed under the prism of *sleep quality*, which refers to *objective* characteristics of good sleep, such as such as ease and time needed to fall asleep, hours of sleep, and physical symptoms during sleep and upon awakening. However, sleep can be experienced as *satisfying* even in the absence of objective quality components; for example, one may feel satisfied with only a few hours of uninterrupted sleep, while someone else may feel unsatisfied with long hours of sleep that are restless. Therefore, sleep quality is related but different to sleep satisfaction, and emerging empirical efforts aim to disentangle sleep quality (and its measurement) from sleep satisfaction (and its measurement) emerging. Building on this emerging research, we will develop and validate a novel sleep satisfaction self-report measure, the Suffolk Sleep Index (SuSI), targeted to an English population. To that aim, we will employ a community-based participatory research (CBPR) approach, partnering with a local charity and involving the Suffolk community in the development of the SuSI.

This research will result in the first sleep satisfaction self-report measure and manual, targeted to the needs and characteristics of the Suffolk community. The SuSI developmental process will also provide a record of Suffolk community members’ conceptualization of sleep satisfaction, including an assessment of their sleep characteristics and satisfaction, with related health indices.

The protocol presented here, and the instrument resulting from it, have important implications for research and practice. In terms of research, the protocol can form a template to guide the development of other similar instruments. Once developed, the SuSI would be used to measure sleep satisfaction, in sleep and health-related studies across disciplines. The SuSI could also be tested for its usability and appropriateness in other populations and cultures. In terms of practice, the SuSI will be suitable for use in a variety of applied health settings. It may be used by health practitioners to assess patient/client satisfaction with sleep as a standalone instrument, or alongside other sleep and health measures. Consequently, we expect that the SuSI protocol and resulting instrument to contribute substantially to advancing knowledge on sleep satisfaction conceptualization and measurement, with strong relevance for sleep (satisfaction) and health-related research and practice.

## Ethics statement

The present study was reviewed and approved by the University of Suffolk Research Ethics Committee (approval ID # RETH(S)21/051). The study adheres to all ethical standards relating to research with human participants, as put forth by the American Psychological Association (American Psychological Association, 2016) and the British Psychological Society (Oates et al., 2021). All participants will be offered information sheets, informed consent sheets, and debriefing sheets. Participation will depend on written informed consent.

## Author contributions

CP: conceived the research, designed the study, developed the materials, wrote the original protocol manuscript, and acts as project lead and administrator. CM: provided input on study design, reviewed and edited the manuscript. VG: reviewed and edited the manuscript. VG: provided input on study design, secured funding. All authors contributed to the article and approved the submitted version.

## Funding

The study has received funding by the National Health Service (NHS) Ipswich and East Suffolk Clinical Commissioning Group (grant number: RD22058). The funding includes publication fees.

## Conflict of interest

The authors declare that the research was conducted in the absence of any commercial or financial relationships that could be construed as a potential conflict of interest.

## Publisher’s note

All claims expressed in this article are solely those of the authors and do not necessarily represent those of their affiliated organizations, or those of the publisher, the editors and the reviewers. Any product that may be evaluated in this article, or claim that may be made by its manufacturer, is not guaranteed or endorsed by the publisher.

## Footnotes

^1^Study materials are available on the OSF platform: https://osf.io/wbsnv/.

^2^https://www.questionpro.com/
